# Mucosal Immune Responses in People Living with HIV May Confer Protection from SARS-CoV-2 Infections After COVID-19 Vaccination

**DOI:** 10.3390/vaccines14060493

**Published:** 2026-05-30

**Authors:** Albert Judith, Muruganantham Lillimary Eniya, Beulah Faith, Poongulali Selvamuthu, Ramamurthy Silamban Yazhini, Nagalingeswaran Kumarasamy, Stephen J. Challacombe, Priya Kannian

**Affiliations:** 1 VHS Laboratory Services, The Voluntary Health Services, Chennai 600113, India; judithalbert1998@gmail.com (A.J.); enianand98@gmail.com (M.L.E.); yazhinisilamban3333@gmail.com (R.S.Y.); 2 Infectious Diseases Medical Centre, The Voluntary Health Services, Chennai 600113, India; beulah@cartcrs.org (B.F.); poongulali@cartcrs.org (P.S.); kumarasamy@cartcrs.org (N.K.); 3 Faculty of Dentistry, Oral & Craniofacial Sciences, King’s College London, London WC2R 2LS, UK; stephen.challacombe@kcl.ac.uk

**Keywords:** mucosal immunity, PLWH, COVID-19 vaccination, secretory IgA antibodies, stimulated oral fluid, blood

## Abstract

**Background/Objectives**: The induction of anti-SARS-CoV-2 antibodies by COVID-19 vaccination reduces morbidity and mortality, but immune responses may be compromised in people living with HIV (PLWH). The aims of the current study were to determine whether viral suppression (VS) or immune reconstitution (IR) in PLWH directly affected their ability to produce effective levels of anti-SARS-CoV-2 antibodies in mucosal secretions or blood induced by vaccination. **Methods**: Anti-SARS-CoV-2 spike IgG, IgA and secretory IgA (SIgA) antibodies and their avidities were measured by ELISA in HIV-negative healthy controls (HC; n = 49) and PLWH (n = 94) using stimulated oral fluid (SOF) and serum. Frequencies of CD4/CD8 T cells and their expression of exhaustion/senescence were determined by flow cytometry. Cytokine levels were measured by cytokine bead arrays. **Results**: We showed that higher HIV burden negatively impacted the levels of systemic and mucosal anti-SARS-CoV-2 spike IgG antibodies produced. This differential IgG antibody production was unaffected by IR status, antiretroviral therapy duration or T cell exhaustion/senescence. PLWH elicited higher anti-SARS-CoV-2 spike IgA antibodies both in peripheral blood and oral mucosa and highr secretory IgA (SIgA) antibodies in the oral mucosa. PLWH with higher HIV RNA copies elicited lower IgG avidity but the IgA avidity indices remained unaffected. PLWH expressed higher levels of innate immunity cytokines in the oral mucosa, irrespective of the HIV RNA copies. **Conclusions**: Significantly fewer breakthrough infections in PLWH compared with HC, along with high IgA/SIgA antibodies and increased innate immunity cytokines in the SOF, suggest a potential role for mucosal immunity in the immunopathogenesis of COVID-19.

## 1. Introduction

The induction of anti-SARS-CoV-2 antibodies by COVID-19 vaccination reduces morbidity and mortality [1,2,3,4]. Antibodies induced by vaccination are primarily of the IgG isotype. T cell-dependent B cell activation is very important for early class switching from the initial IgM to IgG isotype [5]. CD4 T cells are the main drivers of this process. People living with HIV (PLWH), especially if untreated, are known to have a low CD4 T cell count since these cells are the primary targets of HIV. However, in recent years, PLWH on long-term antiretroviral therapy (ART) usually have >500 CD4 T cells/µL [6,7]. Once the HIV RNA copies are reduced to below 20–30 copies/mL or to undetectable levels by ART, a state of viral suppression (VS) is attained. This state of VS facilitates the immune reconstitution (IR) of T cells, which results in normal or near-normal levels of CD4 T cells. Even when CD4 T cell counts are near normal, these CD4 T cells exhibit exhaustion or senescence due to the chronic immune activation state in PLWH [8,9]. T cell exhaustion is a reversible phenomenon where the T cells cannot proliferate but they can produce cytokines. T cell senescence is an irreversible phenomenon where the T cells can neither proliferate nor produce cytokines [10].

In PLWH, the mucosal layers maintain a chronic immune activation state in order to keep the viral burden low or to minimize opportunistic infections [9,11,12]. In the case of COVID-19, SARS-CoV-2 is also known to infect the epithelial cells and minor salivary glands of the oral mucosa [12,13]. During the first and second COVID-19 waves, the majority of PLWH on ART developed moderate COVID-19 [3,14] compared with their age-matched HIV-negative controls (HC), who developed mild COVID-19 [4,15]. Several studies have shown that PLWH with CD4 T cell counts less than 200–300 cells/µL are at higher risk of greater morbidity [16,17]. PLWH are at higher risk of developing more severe outcomes/hospitalization with COVID-19 [18,19].

With the initiation of COVID-19 vaccination programmes, several investigators have addressed the issue of the elicitation of anti-SARS-CoV-2 antibodies in PLWH post vaccination. Lombardi et al. showed that equivalent levels of anti-SARS-CoV-2 antibodies were elicited by PLWH compared with the HC cohorts [20]. These anti-SARS-CoV-2 antibodies appear to be functional since PLWH have been shown to be protected from COVID-19 severity upon vaccination [21]. Taken together these findings suggest that vaccination in PLWH should protect against or reduce the severity of opportunistic infections. However, this suggestion may be dependent on VS and/or IR status in PLWH.

The aims of the current study were to determine whether VS or IR in PLWH directly affected their ability to produce effective and functional levels of anti-SARS-CoV-2 spike IgG, IgA and secretory IgA (SIgA) antibodies in the peripheral circulation and the oral mucosa after COVID-19 vaccination.

## 2. Methods

### 2.1. Patients and Samples

This study was approved by the Voluntary Health Services–IEC (VHS-IEC; proposal#: VHS-IEC/87-2021). All participants provided voluntary written informed consent. Demographics and detailed medical history were collected from all participants (n = 143). HIV-negative healthy controls (HC; n = 49) and people living with HIV (PLWH; n = 94) were recruited from the Voluntary Health Services (VHSs), Multi-Speciality Hospital & Research Institute at 6–12 months after 2–3 COVID-19 vaccinations. Participants received either Covishield or Covaxin for the whole of their vaccination schedule without any mixing. The PLWH cohort was divided into five groups based on their VS (HIV RNA ≤20 copies/mL) and IR (CD4 T cell count >200 cells/µL) status—(1) VS-IR: PLWH with both VS and IR (n = 54); (2) VS-NoIR: PLWH with VS and without apparent IR (CD4 T cell count ≤200 cells/µL; n = 2); (3) NoVS-IR: PLWH without VS (HIV burden >1000 copies/mL) but with IR (n = 16); (4) NoVS-NoIR: PLWH without VS and without IR (n = 9); (5) LoVL-IR: PLWH with a low viral load (20–1000 copies/mL) and with IR (n = 13).

Peripheral blood and stimulated oral fluid (SOF) samples were collected and processed as described previously [22]. Sera were stored at −80 °C until further use. Peripheral blood mononuclear cells (PBMCs) were isolated using Ficol gradient (HiMedia, Mumbai, India) and were used for flow cytometry, and any remaining cells were stored in 10% Dimethyl Sulphoxide (DMSO; HiMedia, India) in liquid nitrogen for further analysis, as described previously [22]. The total volumes of the wax-stimulated SOF samples and the time taken for collection of over 2 mL fluid were noted so that the salivary secretion rates (SRs) could be calculated (SOF volume divided by the time taken) and expressed as ml/min, as described previously [22]. The SOF supernatant was stored at −80 °C and used later for antibody and cytokine assays.

### 2.2. Assay of SARS-CoV-2 Spike-Specific IgG, IgA and SIgA Antibodies Using ELISA

Serum (dilutions 1:2000 to 1:32,000) and SOF (dilutions 1:2 to 1:400) samples were tested for anti-SARS-CoV-2 spike IgG (IgG1 and IgG2 subclasses) antibodies (referred to henceforth as IgG antibodies) using a commercially available kit (Invitrogen, Carlsbad, CA, USA) as per the manufacturer’s instructions. Serum (dilutions 1:2000 to 1:16,000) and SOF (dilutions 1:20 to 1:160) samples were tested for anti-SARS-CoV-2 spike IgA antibodies (referred to henceforth as IgA antibodies) and SOF (dilutions 1:2 to 1:16) samples were tested for anti-SARS-CoV-2 spike secretory IgA antibodies (referred to henceforth as SIgA antibodies) by using an in-house ELISA. For the detection of SIgA antibodies, an anti-secretory component antibody (Invitrogen, USA) was used, as described in detail previously [2].

### 2.3. Avidity Assay for IgG and IgA Antibodies

For avidity assays, samples with an OD value greater or equal to 0.2 were selected using the above-mentioned IgG or IgA antibody assays. An additional step of incubating samples with 4M Urea (Merck, Kenilworth, NJ, USA) was carried out at RT for 20 min after the normal sample incubation step. The remaining protocol was followed as detailed previously [2]. Samples with OD values greater than 2.0 were diluted appropriately in order to obtain an OD value between 0.2 and 2.0 when tested without urea. The avidity index (AI) was calculated as a percentage by dividing the OD value obtained with 4M urea by the OD value without urea. For validating the reproducibility of the avidity assays, the same samples were tested at two independent time points and the CV for each of the samples was calculated. The average of the CV values was finally calculated.

### 2.4. CD4 T Cell Exhaustion/Senescence Using Flow Cytometry

Surface staining for PBMCs was carried out with mouse anti-human antibodies (BD Biosciences, Franklin Lakes, NJ. USA or BioLegend, San Diego, CA, (v1.5) USA) for flow cytometry, as described previously [22]. The fluorochromes and clones used for the cell markers were CD3 (PerCP mouse anti-human CD3: Clone SK-7), CD4 (APC-Cy7 mouse anti-human CD4: Clone SK-3), PD-1 (BV605 mouse anti-human PD-1: Clone EH12.1), TIM-3 (BB515 mouse anti-human TIM-3: Clone 7D3), CD57 (APC mouse anti-human CD57: Clone NK-1) and KLRG-1 (PE mouse anti-human KLRG-1: Clone 14C2A07). The cells were finally acquired in BD FACS Lyric (BD Biosciences, USA) and analysed using the BD FACS Suite software (v1.5).

### 2.5. Cytokine Assays

SOF and serum samples were assayed for cytokine levels of TGF-β, IL-6, IL-8, IL-1β, MIG, MCP-1 and IP-10 using ELISA (R&D Systems, USA) or cytokine bead arrays (CBAs; BD Biosciences, USA), as described previously [2]. SOF samples were tested at 1:2 dilutions and neat serum samples were tested. The detection limit for the ELISA was 0–2000 pg/mL and for the CBA it was 0–2500 pg/mL. Samples that yielded a value higher than the detection limit were diluted appropriately.

### 2.6. Statistical Analysis

The graphs were generated using GraphPad Prism 8.0. The medians, standard deviations and statistical significances were calculated using GraphPad Prism 8.0 or the free online calculators from Social Science Statistics. Comparisons of the medians in the box and whiskers plots were performed using the Mann–Whitney rank sum U test. The Spearman Rank test was used for correlation analyses and Fisher’s exact test was used for statistical significance. Levene’s test was used to assess the equality of variances between the two major cohorts (HC and PLWH). Comparisons of the cytokine mean values between the cohorts and sample types were performed using Anova testing.

## 3. Results

The demographic details of all the participants are given in Table 1. The mean age of the HC group was 25.8 years and that of the PLWH group was 47.6 years. In both groups, the majority were males and were aged less than 60 years. Since age is a known determinant of vaccine response and mucosal immunity, we performed an age-stratified analysis. There were no significant differences detected when stratified by age within any of the HC or PLWH groups (Supplemental Figure S1). Additionally, Spearman rank correlation between the age and anti-SARS-CoV-2 antibodies showed a statistically significant positive correlation (r = 0.1879; *p* = 0.04). All participants had been vaccinated against SARS-CoV-2 and IgG antibodies were detected in all participants. Covishield (identical to ChAdOx1 and manufactured in India) was the most common vaccine administered to both groups (123/143–86%). The second most common vaccine administered was Covaxin (indigenous inactivated whole-virus vaccine; 19/143–13%). One PLWH participant had taken the Sputnik vaccine, which is also a recombinant adenoviral vector-based SARS-CoV-2 S gene vaccine. No significant differences in the anti-SARS-CoV-2 IgG antibodies in serum were detected when stratified by the type of vaccine administered (Covishield vs. Covaxin) or by the duration in months after the last vaccine dose (data not shown).

### 3.1. PLWH Elicited Higher Anti-SARS-CoV-2 Spike SIgA Antibody Values

**Figure 1:**
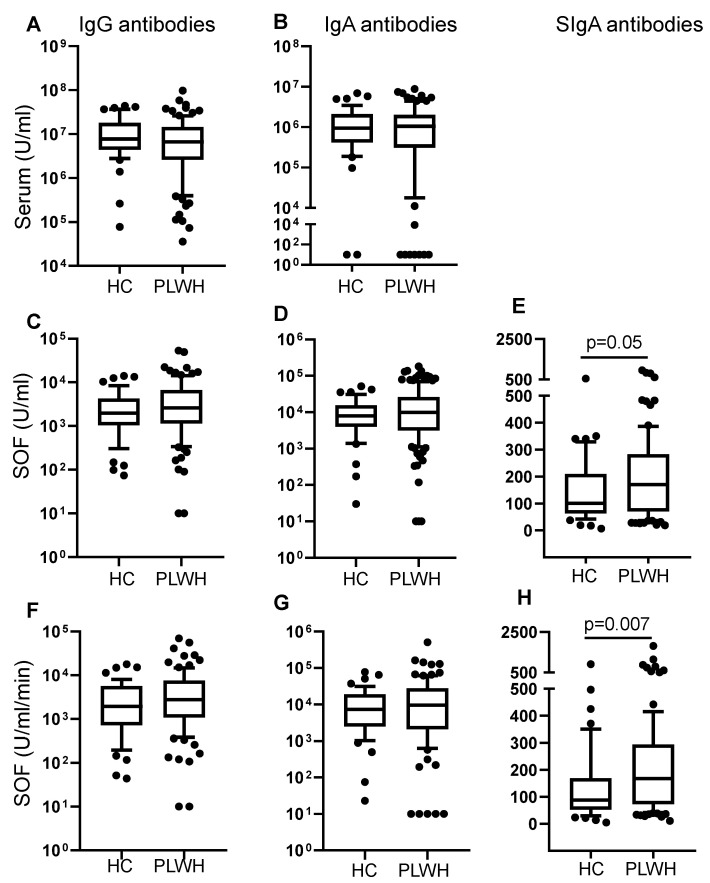
Anti-SARS-CoV-2 spike IgG, IgA and SIgA antibodies in serum and stimulated oral fluid (SOF) after COVID-19 vaccination. PLWH—people living with HIV; HC—HIV-negative healthy controls. The Y-axis denotes the anti-SARS-CoV-2 spike antibodies in logarithmic units/mL. (**A**) IgG antibodies in serum (U/mL). (**B**) IgA antibodies in serum (U/mL). (**C**) IgG antibodies in SOF (U/mL). (**D**) IgA antibodies in SOF (U/mL). (**E**) SIgA antibodies in SOF (U/mL). (**F**) Secretion rate of IgG antibodies in SOF (U/mL/min). (**G**) Secretion rate of IgA antibodies in SOF. (**H**) Secretion rate of SIgA antibodies in SOF. *p* values by Mann–Whitney rank sum U test.

In our ELISAs, serum IgG antibody values (against the standard) appeared to be 6–8-fold greater than IgA antibody values, whilst in SOF samples IgA antibodies to the spike protein were 4–6 times higher than IgG antibodies (Figure 1). No significant differences in the median concentration of serum IgG or serum IgA antibodies were found between HC and PLWH cohorts (Figure 1A,B, Table 2).

Breakthrough infections (BTIs) with SARS-CoV-2 were confirmed by RT-PCR in 8/49 (16%) HC but only in 1/94 (1%) PLWH (*p* = 0.0008; Fisher exact test). The one PLWH participant who developed BTIs belonged to the NoVS-NoIR group.

However, PLWH showed a much greater variation in serum antibody responses to vaccination (*p* < 0.01; Levene’s test). Similarly, in SOF, no significant differences in median IgG or IgA antibodies either in absolute values (units/mL) or the secretion rate (SR) of IgG and IgA antibody (units/mL/min) were apparent (Figure 1C,D,F,G) but IgA antibodies showed a greater variation in PLWH than in HC (Figure 1D,G; *p* < 0.02; Levene’s test). The median level of SIgA antibodies was significantly greater in PLWH than in HC expressed either as units/mL (Figure 1E; *p* < 0.05) or units/mL/min (Figure 1H; *p* < 0.007) The higher SIgA antibodies in the SOF of PLWH suggest significant local antibody production in the oral cavity post COVID-19 vaccination.

### 3.2. Viral Suppression (VS) in PLWH Has a Greater Impact on Antibody Production than Immune Reconstitution (IR)

**Figure 2:**
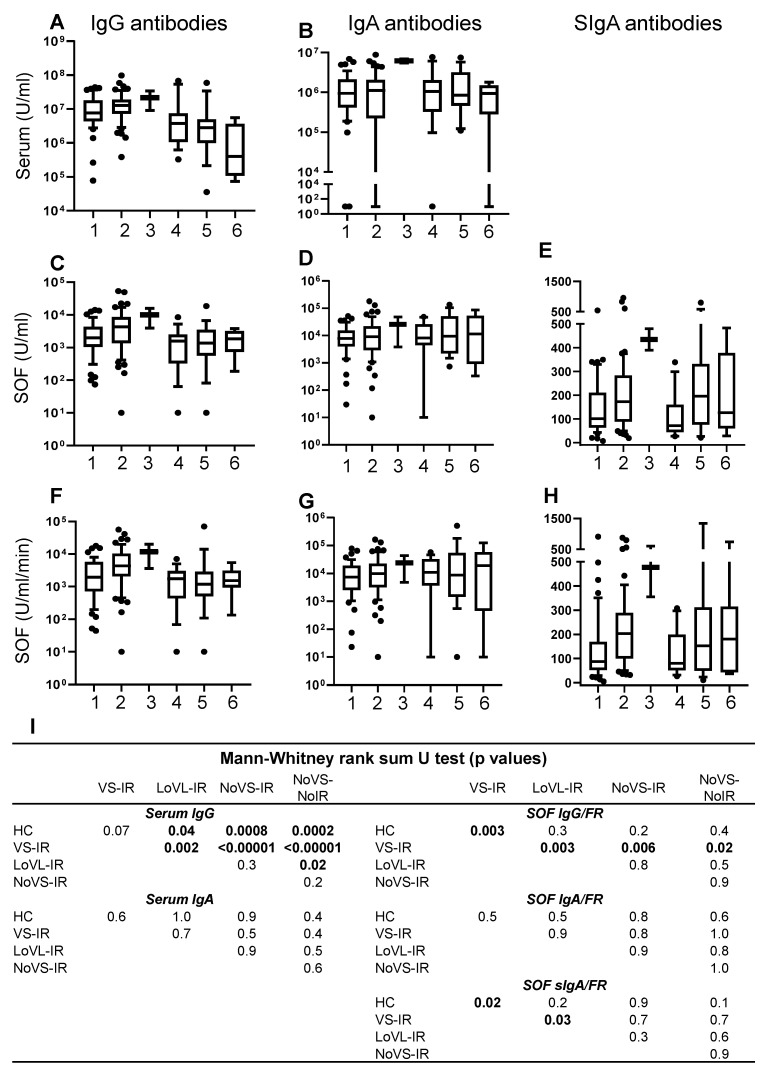
Anti-SARS-CoV-2 spike IgG, IgA and SIgA antibodies in serum and stimulated oral fluid (SOF) after COVID-19 vaccination in relation to viral suppression (VS) and immune reconstitution (IR) in people living with HIV (PLWH). NoIR—without immune reconstitution; NoVS—without viral suppression; LoVL—low viral load. 1: HIV-negative healthy control; 2: PLWH with VS-IR; 3: PLWH with VS-NoIR; 4: PLWH with LoVL-IR; 5: PLWH with NoVS-IR; 6: PLWH with NoVS-NoIR. (**A**) IgG antibodies in serum. (**B**) IgA antibodies in serum. (**C**) IgG antibodies in SOF. (**D**) IgA antibodies in SOF. (**E**) SIgA antibodies in SOF. (**F**) Secretion rate of IgG antibodies in SOF (U/mL/min). (**G**) Secretion rate of IgA antibodies in SOF. (**H**) Secretion rate of SIgA antibodies in SOF. (**I**) Mann–Whitney rank sum U test *p* values comparing different cohorts.

To determine the relative impacts of VS and IR on antibody production, we analysed the post-vaccination antibody levels after categorizing the PLWH into five groups based on their VS and IR status. There was no significant difference in the median values of serum IgG antibodies between VS-IR and HC groups (Figure 2A), but the median IgG antibody level in the NoVS-NoIR group was significantly less than that of the other groups (*p* < 0.002; Figure 2A). There was no statistically significant difference in the median serum IgA antibodies after vaccination but both VS-IR and NoVS-NoIR groups showed a wide variation in responses (Figure 2B).

In SOF, the median IgG antibody level was raised in VS-IR compared with HC (*p* = 0.003; Figure 2C,F) but no significant differences were apparent with median IgA antibodies (Figure 2D,G). The lowest SIgA antibodies were found in the LoVL-IR group, which was significantly different from the VS-IR group (*p* = 0.03). The SR of SIgA antibodies was greater in the VS-IR group (Figure 2H) than in HC (*p* = 0.02) and the LoVL-IR (*p* = 0.03) group, but no clear differences were found in other groups (Figure 2E,H). These findings suggest that the elicitation of IgG antibodies is impacted by VS/IR status but that IgA antibodies remain unaffected. The statistically significant differences are given in the table inset in Figure 2I. PLWH with VS-NoIR have a low or undetectable number of HIV RNA copies, but their immune system has not been able to attain CD4 T cell counts above 200 cells/µL. This is a rare scenario, affecting only two participants. Therefore, this group was not included in any of the statistical analyses performed in this study.

**Figure 3:**
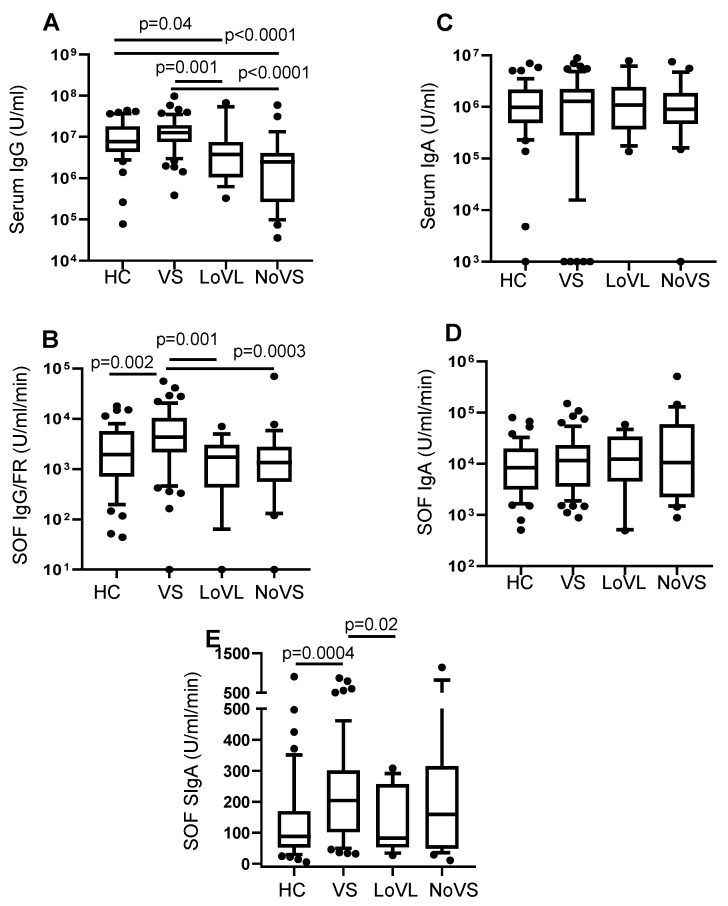
Relationship between HIV viral load and anti-SARS-CoV-2 spike IgG antibodies in serum and stimulated oral fluid (SOF) samples after COVID-19 vaccination. VS = viral suppression; NoVS = without viral suppression; LoVL= low viral load. (**A**) IgG antibodies in serum. (**B**) Secretion rate of IgG antibodies in SOF (U/mL/min). (**C**) IgA antibodies in serum. (**D**) Secretion rate of IgA antibodies in SOF. (**E**) Secretion rate of SIgA antibodies in SOF. *p* values by Mann–Whitney rank sum U test.

Since serum IgG antibodies to the spike protein after vaccination appeared to be related to viral suppression of HIV (Figure 2), PLWH cohorts were stratified according to their viral loads irrespective of their IR status. Median serum IgG antibodies in the NoVS group were significantly lower than those in the VS group and the HC group (*p* < 0.0001; Figure 3A). In contrast, no significant differences in median serum IgA antibody levels were found (Figure 3C). In SOF, the median SR of IgG antibodies in the VS group was greater than that in the HC (*p* = 0.002), LoVL and NoVS groups (*p* < 0.001; Figure 3B). No differences were found in the SR of IgA antibodies in SOF. (Figure 3D). However, for SIgA antibodies in SOF, the secretion rate per min was greater in the VS group than in HC (*p* = 0.0004; Figure 3E).

The median antibody levels and their ratios in the HC, VS, LoVL and NoVS groups are given in Table 2. Serum IgG/IgA ratios were significantly smaller in the NoVS group compared with the HC (*p* = 0.0002) and VS (*p* < 0.00001) groups, and also between VS and LoVL groups (*p* = 0.02). Similarly lower SOF IgG/SIgA ratios were evidenced in the NoVS group compared with the HC (*p* = 0.04) and VS (*p* = 0.005) groups (Table 2). The NoVS group had higher ratios of serum/SOF IgG antibodies compared to the HC (*p* = 0.00008) and VS (*p* = 0.004) groups. Taken together, our findings suggest that following SARS-CoV-2 vaccination, PLWH with high HIV RNA copies elicit lower serum and SOF IgG antibodies but those with effective suppression of HIV elicit higher serum IgG and SIgA antibodies than HC and the other PLWH groups.

### 3.3. Factors Possibly Influencing SARS-CoV-2-Specific Antibody Production

**Figure 4:**
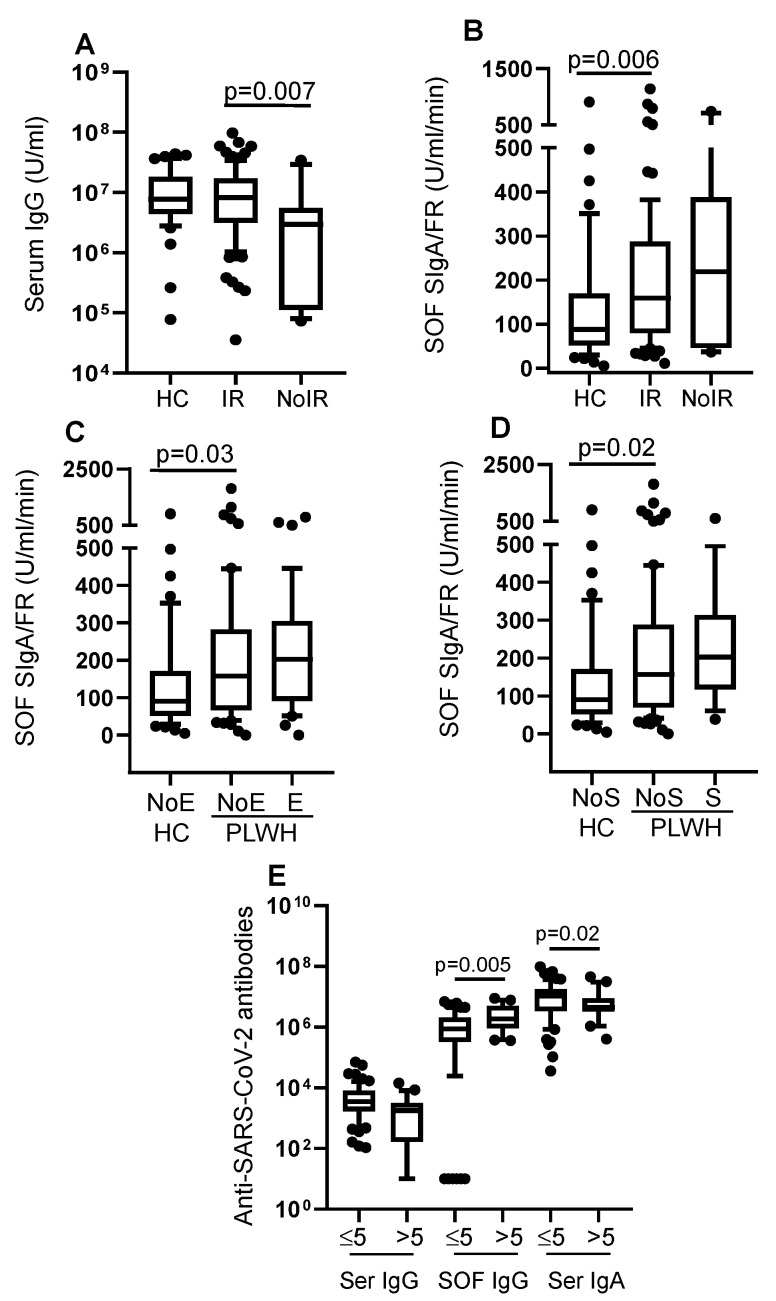
The effect of immune reconstitution (IR), T cell exhaustion, T cell senescence and the duration of antiretroviral therapy (ART) on antibody responses in serum and stimulated oral fluid (SOF). NoIR = without immune reconstitution; NoE = no CD4 T cell exhaustion; E = CD4 T cell exhaustion; NoS = no CD4 T cell senescence; S = CD4 T cell senescence. PLWH stratified based on IR—(**A**) serum IgG antibodies; (**B**) SIgA antibodies in SOF (secretion rate). PLWH stratified based on CD4 T cell exhaustion—(**C**) SIgA antibodies in SOF (secretion rate). PLWH stratified based on CD4 T cell senescence—(**D**) SIgA antibodies in SOF (secretion rate). PLWH stratified based on the duration of ART—(**E**) snti-SARS-CoV-2 spike antibodies in serum or in SOF. *p* values by Mann–Whitney rank sum U test.

**Immune reconstitution:** We stratified PLWH cohorts based on their IR status (IR or NoIR) and compared the various anti-SARS-CoV-2-specific antibody levels irrespective of their VS status. While serum IgG antibody production in the IR group was similar to that of the controls (HC), that of the NoIR group was significantly less than that of the IR group (*p* = 0.007; Figure 4A). In SOF, the median SIgA antibodies was greater in the IR group than in HC (*p* = 0.006; Figure 4B). No significant differences were noted among the IR and NoIR groups for serum IgA antibodies or SOF IgG and IgA antibodies.

**Peripheral blood CD4 T cell senescence and exhaustion**: The PLWH cohort was also stratified based on their CD4 T cell exhaustion or senescence levels. The mean + 2SD of the frequency values of CD4 T cell exhaustion/senescence in the HC group (7%) was used as the cut-off to stratify those with and without CD4 T cell exhaustion/senescence. CD4 T cell exhaustion was detected in 32/94 (34%) and CD4 T cell senescence was detected in 12/94 (13%) of the PLWH. Only one HC participant showed CD4 T cell exhaustion/senescence above the fixed cut-off (>7%). Median values of SIgA antibodies to the spike antigen in the PLWH groups was slightly higher than that in HC irrespective of exhaustion (Figure 4C) or senescence (Figure 4D). There were no differences in the median values of serum and SOF IgG and IgA antibodies.

**Time since ART**: The PLWH cohort was stratified into those on ART for less than five years or more than five years, independently from VS and IR status. Median serum IgG, SOF IgA and SOF SIgA antibodies were similar in both groups. SOF IgG antibodies were significantly lower (*p* = 0.005) and serum IgA antibodies were significantly higher (*p* = 0.02) in PLWH who had been on ART for more than five years (Figure 4E).

### 3.4. IgA and SIgA Antibodies Are Elicited in the Oral Mucosa of PLWH upon Vaccination

**Figure 5:**
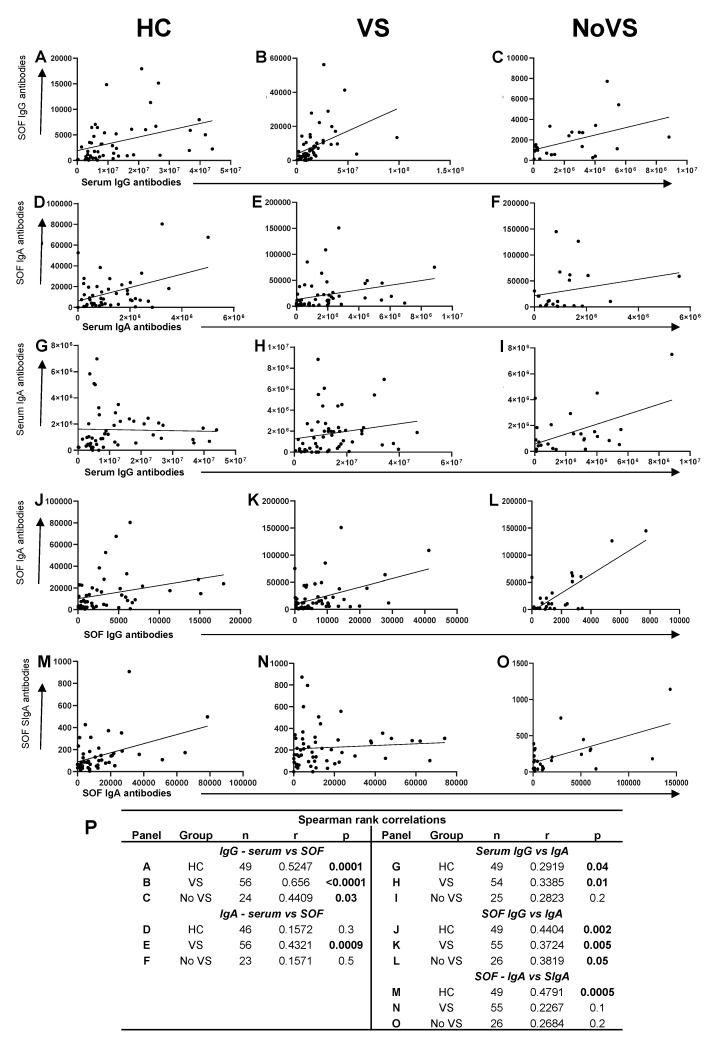
Correlations between systemic and mucosal IgG and IgA antibodies in HIV-negative healthy controls (HC) and people living with HIV (PLWH). Serum antibody levels are expressed in U/mL. Secretion rates of stimulated oral fluid (SOF) antibodies are expressed in U/mL/min. VS = PLWH with viral suppression; NoVS = PLWH without viral suppression; Serum IgG vs SOF IgG antibodies (**A**–**C**); Serum IgA vs SOF IgA antibodies (**D**–**F**); Serum IgG vs serum IgA antibodies (**G**–**I**); SOF IgG vs SOF IgA antibodies (**J**–**L**); SOF IgA vs SOF SIgA antibodies (**M**–**O**); Spearman rank correlations (**P**). P = correlation coefficient; *p* value by Spearman rank.

IgG, IgA and SIgA antibodies in serum and SOF were correlated with each other in the three groups—HC, VS and NoVS (Figure 5)—and the statistical values of r and *p* were calculated using Spearman rank test (Figure 5P). There were statistically significant positive correlations between serum IgG antibodies and SOF IgG antibodies in HC (*p* = 0.0001; Figure 5A), PLWH with VS (*p* < 0.0001; Figure 5B), and PLWH with NoVS (*p* = 0.03; Figure 5C). Statistically significant positive correlations were seen between serum and SOF IgA antibodies in PLWH with VS (*p* = 0.0009; Figure 5E) but not in the HC or NoVS groups. Positive correlations between IgG and IgA antibodies in SOF were found in all three groups (Figure 5J–L). Serum IgG and IgA antibodies were significantly correlated in the HC and VS groups, but not in the NoVS group (Figure 5G–I). SIgA antibodies were significantly correlated with IgA antibodies in HC but not in the VS or NoVS group (Figure 5M–O). The Spearman rank correlation r values and their corresponding *p* values are shown in the table inset (Figure 5P).

### 3.5. Differential IgG Avidities and Similar IgA Avidities

**Figure 6:**
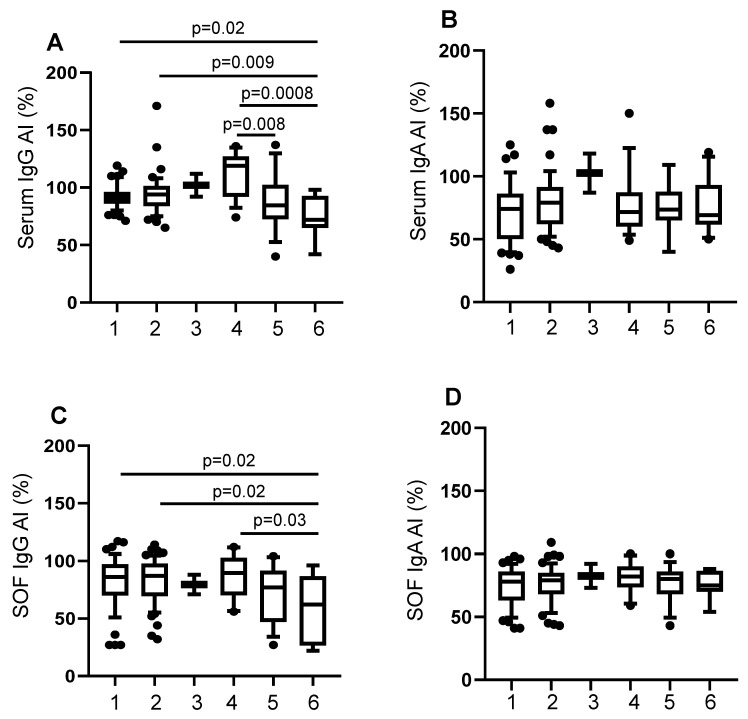
Avidity indices (AI) of IgG and IgA anti-SARS-CoV-2 antibodies in serum and stimulated oral fluid (SOF) samples (median ± range). 1: Healthy controls (HC); 2: PLWH with VS-IR; 3: PLWH with VS-NoIR; 4: PLWH with LoVL-IR; 5: PLWH with NoVS-IR; 6: PLWH with NoVS-NoIR. The Y-axis denotes the avidity indices in percentages of control (%). (**A**) Serum IgG avidity index. (**B**) Serum IgA avidity index. (**C**) SOF IgG avidity index. (**D**) SOF IgA avidity index. *p* values by Mann–Whitney rank sum U test.

IgG and IgA antibodies were tested for their avidity indices using 4M urea to dissociate antibody binding. The reproducibility of these avidity assays was tested with 80 serum and 12 SOF samples for IgG antibodies, and 20 serum and SOF samples each for IgA antibodies. The coefficient of variation was satisfactory (serum and SOF IgG antibody avidity: 3%; serum and SOF IgA antibody avidity: 5%). The IgG avidity indices were similar in the HC and PLWH with VS or LoVL both in serum and SOF samples (Figure 6A,C). The serum and SOF IgG avidity indices were significantly lower in the two NoVS groups than in the other groups (*p* < 0.02; Figure 6A,C). In contrast, no significant differences in serum or SOF IgA antibody avidity between the groups were seen. (Figure 6B,D). Thus, PLWH with higher HIV RNA copies elicit lower IgG antibodies that also have lower binding capacities, while both the levels and the avidity indices of IgA antibodies remain unaffected.

### 3.6. Higher Expression of Innate Immunity Cytokines in the Oral Mucosa of PLWH

**Figure 7:**
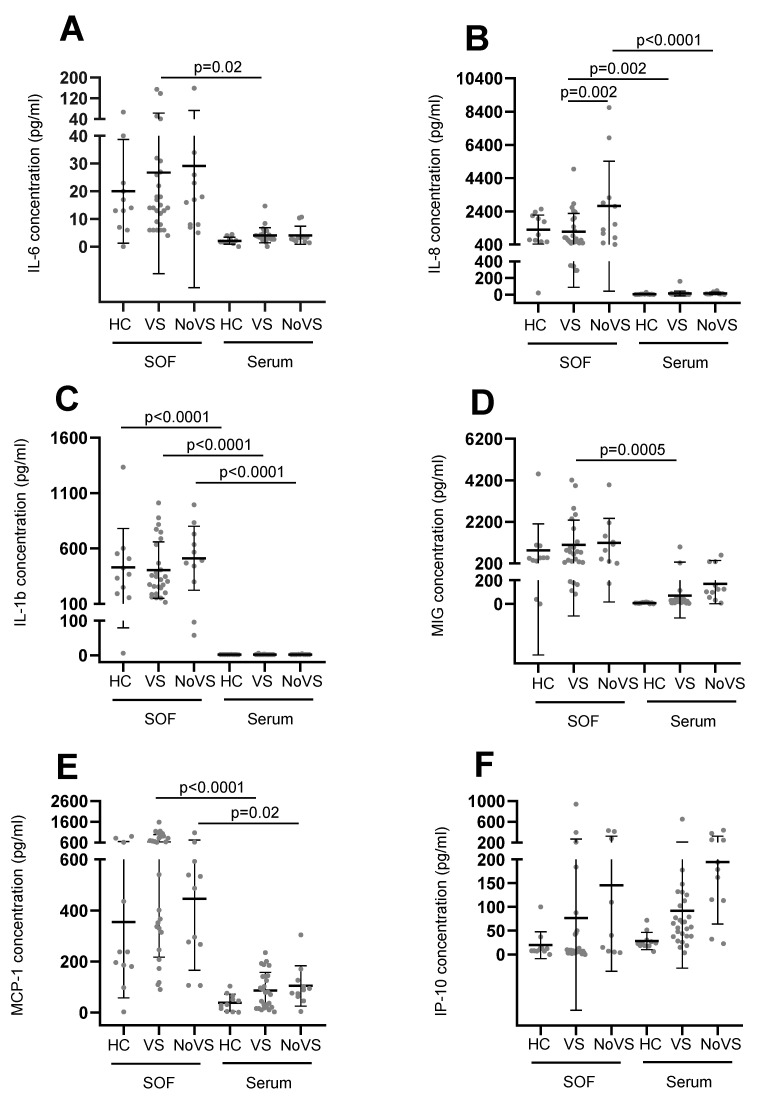
Higher expression of innate immunity cytokines in the oral mucosa of people living with HIV (PLWH). HC = healthy control; VS = PLWH with viral suppression; NoVS= PLWH without viral suppression; Y-axis = cytokine concentrations (pg/mL). The black bars denote the mean values and the error bars denote the standard deviations. (**A**) IL-6; (**B**) IL-8; (**C**) IL-1β; (**D**) MIG; (**E**) MCP-1; (**F**) IP-10. *p* values by Anova.

Innate immunity cytokines—IL-6, IL-8, IL-1β, MIG, MCP-1 and IP-10—were expressed in detectable levels in both SOF and serum in all groups (Figure 7). These cytokine levels were significantly higher in the SOF compared with serum, except IP-10. Although the VS and NoVS PLWH groups expressed higher levels of these cytokines in the SOF compared to the HC group, statistical significance was reached only for IL-8. In serum, there were marked differences between the HC and PLWH groups only for MIG, MCP-1 and IP-10. Taken together, our findings suggest that the PLWH expressed higher levels of innate immunity cytokines irrespective of HIV RNA copies in the oral mucosa.

## 4. Discussion

PLWH were vaccinated as high priority when the COVID-19 vaccination programme was implemented because of their immunocompromised state. An underlying question always exists as to whether PLWH would be able to mount the desired antibody response upon vaccination. In this study, we evaluated the anti-SARS-CoV-2 spike IgG, IgA and SIgA antibody responses 6–12 months after two to three doses of COVID-19 vaccination in HC and in PLWH who were stratified based on VS and IR status. PLWH were able to elicit an almost similar anti-SARS-CoV-2-specific antibody response as the HC in both the peripheral circulation and the oral mucosa. The majority of the participants in both the HC and PLWH groups were below 60 years of age. However, the median ages of these groups were significantly different, but age-wise stratification did not show any significant differences in the IgG antibody levels in this study cohort. A positive correlation of age and antibody levels irrespective of the study cohorts suggests that age might not impact the levels of anti-SARS-CoV-2 antibodies analysed in this study. High HIV RNA copienegatively impacted the elicitation of serum and SOF IgG antibodies, while the IgA antibody response appeared unaffected. In PLWH cohorts, the median SIgA antibody responses were unexpectedly higher than in the HC. This higher mucosal antibody response might be associated with lower BTIs in PLWH (1%) compared with HC (16%; *p* < 0.001). This significant difference in the rate of BTIs could also be influenced by the behavioural shielding of the PLWH cohort since they are an immunocompromised group compared with HC, considering the collection of these samples during the COVID-19 lock down period. It is interesting to note that other studies have shown lower BTI rates among PLWH post COVID-19 vaccination when compared with HIV-negative counterparts [23,24].

When the PLWH cohort was compared with HC, the IgG and IgA anti-SARS-CoV-2 spike antibodies in serum and SOF were similar, corroborating the findings of another study by Lombardi et al. [20]. Additionally, we showed that SOF SIgA anti-spike antibodies were significantly higher in PLWH compared with HC. Our findings in the PLWH cohort showed a high inter-group variation in the levels of IgG and IgA antibodies as confirmed by Levene’s test. This warranted subsequent stratification of the PLWH group by VS and IR status. When PLWH were further stratified by VS and IR status, IgG antibody levels were higher in HC and PLWH with VS groups both in serum and SOF compared to the LoVL and NoVS groups. This trend was evidenced irrespective of their IR status. This suggests that HIV suppression but not IR status negatively impacts the production of IgG antibodies. Similar findings in the serum samples have been shown in other studies [20,21]. A positive correlation of the IgG antibodies between the serum and SOF suggests that the majority of the IgG antibodies in the SOF comes passively from the peripheral circulation through the gingival crevicular spaces. This is similar to the data we and others had shown previously in a larger HIV-negative cohort after COVID-19 vaccination [2,25]. We also note that IgG subclass responses can be altered in PLWH [26]. Although both IgG1 and IgG2 were detected by our methodology, it may be fruitful to further analyse IgG subclass responses in this cohort.

An important finding was that median SIgA antibody levels in the SOF of all the PLWH groups were greater than in the HC group. These findings suggest a stronger mucosal antibody response to COVID-19 vaccination in PLWH. IgG class switching requires T cell activation, while IgA class switching occurs both in T cell-dependent and T cell-independent manners [27]. Thus, the significantly lower serum IgG antibody levels in the PLWH with NoIR compared to those with IR may be attributed to the T cell dependency for IgG class switching. Elsewhere, it has been shown that circulating high TGF-β levels in PLWH decreases IgG antibody production without impacting IgA antibody production [28]. In our study, TGF- β levels were similar in the HC and PLWH groups both in serum and SOF samples (Supplemental Figures S2 and S3). Proportionate IgA antibody levels among all the groups could therefore be attributed to the similar levels of serum TGF-β. Cognasse et al. have shown that PLWH elicit higher serum IgA antibodies due to the presence of high levels of gp160 antigen [29] and PLWH with LoVL and NoVS are likely to have higher circulating gp160 antigen levels. It has also been shown that serum total IgA levels increase in PLWH due to polyclonal B cell activation [30].

The host immune system in PLWH maintains a chronic immune activation state in order to keepthe episodes of opportunistic infections minimal and also to control the HIV RNA copies. This chronic immune activation state could be directly responsible for the higher polyclonal B cell activation. Navas t al. have elaborated that PLWH express a unique B cell profile characterized by higher abundances of IgA^+^ switched B cells, which leads to improved mucosal immunity and better viral control. Additionally, IgA antibody responses have been shown to trigger a good innate immune response [31]. In this study, we have shown that PLWH expressed higher innate immunity cytokines in the SOF samples. Studies have also shown that monomeric IgA antibodies against SARS-CoV-2 have neutralization capacity [32,33], while oral SIgA antibodies have been shown to be associated with early clearance of the virus [34]. Thus, there are many physiological factors demonstrating a potential role for IgA and SIgA antibody levels in the oral mucosa of PLWH that might be associated with lower subsequent BTIs.

The avidity index (AI) determines the binding capacity of the virus-specific antibodies to the SARS-CoV-2 spike protein. It is known that IgG antibody avidity increases over time as well as with subsequent exposure due to maturation. This is an indication of long-term immunity [35]. The AI may be related to the function of the antibodies since it has been shown that a low IgG antibody AI was associated with an increased chance of acquiring severe COVID-19, while a high IgG antibody AI was more associated with the development of mild COVID-19 during subsequent exposures [36]. In our study, serum IgG antibody AIs were ≥50% in all nine participants who developed mild COVID-19. Among the others who did not develop BTIs, only 3/49 (6%) HC and 6/94 (6%) PLWH had an IgG antibody AI <40%. Interestingly, the NoVS groups, which had low IgG antibody levels, also showed a lower IgG antibody AI in both serum and SOF. In contrast, the serum and SOF IgA antibody avidity was quite similar in all groups including the HC. Thus, in our study, the low IgG antibody AI did not appear to impact the acquisition of SARS-CoV-2 infection or subsequent disease severity.

We analysed a number of other factors that could impact antibody production in PLWH post COVID-19 vaccination—IR status, CD4 T cell exhaustion/senescence and ART duration. Our findings suggest that PLWH with IR elicited higher IgG antibodies in both serum and mucosa compared to those without IR. However, IR status did not appear to impact mucosal (IgA/SIgA) antibody production. Studies have shown that due to the chronicity of immune activation in PLWH, T cells undergo exhaustion and/or senescence [37,38,39]. In our study, mean CD4 T cell exhaustion (34%) and senescence (13%) in PLWH was significantly greater than in HC but did not appear to impact antibody production.

The analysis of IgG, IgA and SIgA antibody levels in serum and SOF based on the duration of ART (less than or more than five years), irrespective of VS and IR status, showed that PLWH who are on ART for more than five years had significantly lower SOF IgG antibodies and higher serum IgA antibodies. The 5-year period was chosen as a long-term timeframe from the initiation of ART because PLWH take about five years to show treatment adherence, successful treatment outcomes with low cumulative probability of treatment failure, and low sub-hazard ratios of several factors that impact treatment outcomes [40,41]. Moreover, Bijker et al. showed that the low sub-hazard ratios were similar at five years and ten years of ART [41]. These findings, taken together, support the conclusion that chronic immune activation in PLWH negatively impacts the serum and mucosal IgG antibody responses but not the IgA antibody responses post COVID-19 vaccination, which may actually be raised, especially in mucosa. We have previously shown that the COVID-19 vaccine elicits innate immunity cytokines in the oral mucosa [2], which might also contribute to the higher IgA antibody responses.

A positive correlation between IgG and IgA anti-spike protein antibodies in serum and SOF would suggest that there is a passive transfer of both IgG and IgA antibodies into the oral cavity from the peripheral circulation. In this study, serum IgG antibodies were not significantly correlated with serum IgA antibodies. However, in SOF, IgA and IgG antibodies were significantly correlated. Since the dimeric SIgA antibody is primarily produced in the mucosae, the results would be consistent with local production of SIgA antibodies.

## 5. Conclusions

A lack of viral suppression does negatively impact the IgG antibody responses of PLWH to vaccination against SARS-CoV-2, but if PLWH have immune reconstitution and viral suppression, their antibody responses are not compromised. In fact, anti-SARS-CoV-2 spike IgA/SIgA antibodies and innate immunity cytokines were raised in the oral mucosa of these PLWH cohorts. These findings, along with the observation that significantly fewer PLWH developed SARS-CoV-2 breakthrough infections, suggest that IgA/SIgA and innate cytokines may play a potential role in mitigating the severity of subsequent COVID-19 episodes. These findings and the lack of association with CD4 T cell counts suggest a potential role for mucosal innate immunity in the immunopathogenesis of COVID-19 that warrants further in-depth explorations and also opens promising avenues to explore mucosal vaccines.

## Figures and Tables

**Table 1:** Demographic details of HC and PLWH stratified based on viral suppression (VS) and immune reconstitution (IR) status.

Overall		HC	PLWH	NoVS-IR	NoVS-NoIR	VS-IR	VS-NoIR	LowVL-IR
	n=	49	94	16	9	54	2	13
Serum IgG pos		49 (100)	94 (100)	16 (100)	9 (100)	54 (100)	2 (100)	13 (100)
Gender	Males	33 (67)	72 (77)	13 (81)	6 (67)	39 (72)	2 (100)	12 (92)
	Females	14 (29)	22 (23)	3 (19)	3 (33)	15 (28)	0	1 (8)
Age (yrs)	<60	48 (98)	81 (86)	14 (88)	8 (89)	48 (89)	1 (50)	10 (77)
	≥60	1 (2)	13 (14)	2 (12)	1 (11)	6 (11)	1 (50)	3 (23)
Vaccine type	Covishield	37 (76)	86 (91)	16 (100)	9 (100)	47 (87)	2 (100)	12 (92)
	Covaxin	12 (24)	7 (7)	0	0	6 (11)	0	1 (8)
	Sputnik	0	1 (1)	0	0	1 (2)	0	0
COVID-19 BTI	Total	8 (16)	1 (1)	0	1 (11)	0	0	0
Gender	Males	6 (75)	1 (100)		1 (100)			
	Females	2 (25)	0		0			
Age (yrs)	<60	8 (100)	1 (100)		1 (100)			
	≥60	0	0		0			
Vaccine type	Covishield	7 (19)	1 (1)		1 (11)			
	Covaxin	1 (8)	0		0			

Percentage of total number in group shown in brackets. BTI = breakthrough infection with SARS-CoV2.

**Table 2:** Median antibody levels to SARS-CoV2 spike protein after vaccination and median antibody ratios among healthy controls (HC) and people living with HIV (PLWH) stratified based on HIV burden.

Sample/Antibody Type	HC		PLWH		
		Overall	VS	LoVL	NoVS
Antibody levels—median					
Serum IgG (U/mL)	7.8 × 10^6^	6.6 × 10^6^	1.3 × 10^7^	3.8 × 10^6^	2.5 × 10^6^
SOF IgG (U/mL/min)	1935	2757	4325	1729	1349
Serum IgA (U/mL)	9 × 10^5^	1 × 10^6^	1.3 × 10^6^	1.1 × 10^6^	8.9 × 10^5^
SOF IgA (U/mL/min)	7373	9576	11,505	12,364	1.630
SOF SIgA (U/mL/min)	88	168	204	83	159
Median antibody ratios					
Serum IgG/IgA	8 *	6	10 #^	**3 #**	2 *^
SOF IgG/IgA	0.3	0.4	0.6	0.1	0.1
SOF IgG/SIgA	22 *	20	27 ^	**16**	11 *^
SOF IgA/SIgA	79	64	41	117	118

Statistically significant differences in the antibody median ratios according to Mann–Whitney rank sum U test. * HC vs. NoVS: *p* = 0.0002 (serum IgG/IgA); *p* = 0.04 (SOF IgG/SIgA). # VS vs. LoVL: *p* = 0.02 (serum IgG/IgA). ^ VS vs. NoVS: *p* < 0.00001 (serum IgG/IgA); *p* = 0.005 (SOF IgG/SIgA). SOF—stimulated oral fluid; U = unit.

## Data Availability

All data pertaining to this article are included here.

## Supplementary Materials

The following supporting information can be downloaded at https://www.mdpi.com/article/10.3390/vaccines14060493/s1: Figure S1: Age-wise stratification of serum anti-SARS-CoV-2 spike IgG antibodies. The circles denote the samples and the error bars denote the mean ± SD. HC—HIV-negative healthy control; VS-IR—PLWH with viral suppression and immunological reconstitution; NoVS—PLWH without viral suppression. Figure S2: Serum TGF-β levels (pg/mL) in HIV-negative healthy controls (HC) and people living with HIV (PLWH) cohorts. The circles denote the samples and the error bars denote mean ± SD. VS—PLWH with viral suppression; Low VS—PLWH with low HIV RNA copies; NoVS—PLWH without viral suppression. Figure S3: Stimulated oral fluid (SOF) TGF-β levels (pg/mL) in HIV-negative healthy controls (HC) and people living with HIV (PLWH) cohorts. The circles denote the samples and the error bars denote mean±SD. VS—PLWH with viral suppression; Low VS—PLWH with low HIV RNA copies; NoVS—PLWH without viral suppression.

## References

[1] Muthukrishnan J., Vardhan V., Mangalesh S., Koley M., Shankar S., Yadav A.K., Khera A. (2021). Vaccination status and COVID-19 related mortality: A hospital based cross sectional study. Med. J. Armed Forces India.

[2] Kannian P., Eniya M.L., Mahanathi P., Gracemary A., Kumarasamy N., Challacombe S.J. (2025). Impact of COVID-19 on mucosal immunity and antibody responses in COVID vaccinees. Vaccines.

[3] Inan A., Barkay O., Karapinar A., Yilmaz-Karadag F., Aktas S., Bolukcu S. (2025). COVID-19 incidence and factors influencing infection risk among people living with HIV in Türkiye: Is current issue the vaccine hesitancy–opposition?. Can. J. Infect. Dis. Med. Microbiol..

[4] Inzaule S., Silva R., Ford N., Thwin S.S., Wassila J., Zumla A., Doherty M., Diaz J., Bertagnolio S. (2025). Comparative analysis of COVID-19 in-hospital mortality in people living with HIV during SARS-CoV-1 pre-delta, delta and Omicron waves: Data from the WHO global clinical platform. AIDS.

[5] Siegrist C.A. (2018). Vaccine Immunology. Plotkin’s Vaccines.

[6] Fichtenbaum C.J., Malvestutto C.D., Watanabe M.G., Smith E.D., Ribaudo H.J., McCallum S., Fitch K.V., Currier J.S., Diggs M.R., Chu S.M. (2025). Effects of antiretrovirals on major adverse cardiovascular events in the REPRIEVE trial: A longitudinal cohort analysis. Lancet HIV.

[7] Jaimsakul A., Rupasinghe D., Woolley I., Choi J.Y., Templeton D.J., Widhani A., Petoumenos K., Tanuma J., Treat Asia HIV Observational Database (TAHOD) and Australian HIV Observational Database (AHOD) of IeDEA Asia-Pacific (2024). HIV treatment outcomes after 10 years on ART in the TREAT Asia observational database and Australian HIV observational database. J. Acquir. Immune Defic. Syndr..

[8] Vangelov D., Emilova R., Todorova Y., Yancheva N., Dimitrova R., Grigorova L., Alexiev I., Nikolova M. (2025). T-lymphocyte phenotypic and mitochondrial parameters as markers of incomplete immune restoration in people living with HIV+ on long-term cART. Biomedicines.

[9] Watanabe M., Jergovic M., Davidson L., La Fleur B.J., Castaneda Y., Martinez C., Smithey M.J., Stowe B.J., Haddad E.K., Nikolich-Zugich J. (2022). Inflammatory and immune markers in HIV-infected older adults on long-term antiretroviral therapy: Persistent elevation of sCD14 and of proinflammatory effector memory T cells. Aging Cell.

[10] Zhang W., Kong D., Zhang X., Hu L., Nian Y., Shen Z. (2025). T cell aging and exhaustion: Mechanisms and clinical implications. Clin. Immunol..

[11] Tugizov S.M. (2023). Molecular pathogenesis of human immunodeficiency virus-associated disease of oropharyngeal mucosal epithelium. Biomedicines.

[12] Hickman D., Moutsopoulos N.M. (2025). Viral infection and antiviral immunity in the oral cavity. Nat. Rev. Immunol..

[13] Bellocchio L., Dipalma G., Inchingolo A.M., Inchingolo A.D., Ferrante L., Del Vecchio G., Malcangi G., Palermo A., Qeadro A., Inchingolo F. (2023). COVID-19 on oral health: A new bilateral connection for the pandemic. Biomedicines.

[14] Xu N., Shen Y., Huang W., Nie J. (2025). The current status in terms of vaccination for individuals infected with human immunodeficiency virus. Viruses.

[15] Chapman A., Berenbaum F., Curigliano G., Pliakas T., Sheikh A., Abduljawad S. (2025). Risk of severe outcomes from COVID-19 in immunocompromised people during the Omicron era: A systematic review and meta-analysis. Clin. Ther..

[16] Kausalya B., Saravanan S., Pallikkuth S., Pahwah R., Saini S.R., Iqbal S., Solomon S., Murugavel K.G., Poongulali S., Kumarasamy N. (2022). Immune correlates of cardiovascular co-morbidity in HIV infected participants from South India. BMC Immunol..

[17] Balagopal A., Asmuth D.M., Yang W., Campbell T.B., Gupte N., Smeaton L., Kanyama C., Grinsztein B., Santos B., Supparatpinyo K. (2015). Pre-cART elevation of CRP and CD4+ T-cell immune activation associated with HIV clinical progression in a multinational case-cohort study. J. Acquir. Immune Defic. Syndr..

[18] Spinelli M.A., Jones B.L.H., Gandhi M. (2022). COVID-19 outcomes and risk factors among people living with HIV. Curr. HIV/AIDS Rep..

[19] Ssentongo P., Heilbrunn E.S., Ssentongo A.E., Advani S., Chinchilli V.M., Nunez J.J., Du P. (2021). Epidemiology and outcomes of COVID-19 in HIV-infected individuals: A systematic review and meta-analysis. Sci. Rep..

[20] Lombardi A., Butta G.M., Donnici L., Bozzi G., Oggioni M., Bono P., Matera M., Consonni D., Ludovisi S., Muscatello A. (2022). Anti-spike antibodies and neutralizing antibody activity in people living with HIV vaccinated with COVID-19 mRNA-1273 vaccine: A prospective single-centre cohort study. Lancet Reg. Health Eur..

[21] Halenmubieke S., Nuermaimaiti A., Liu X., Chang L., Ji H., Sun H., Yam Y., Xu J., Wang L. (2025). Dynamic monitoring of antibody titres in people living with HIV during Omicron epidemic: Comparison between unvaccinated and vaccinated individuals. BMC Infect. Dis..

[22] Nasab S.D.S., Eniya M.L., Judith A., Clasen F., Faith B., Poongulali S., Gita J.B., Ashok C., Raghavi V., Vedavalli S. (2024). Detection and consistency of mucosal fluid T lymphocyte phenotypes and their relationship with blood, age and gender. J. Immunol. Methods.

[23] Matveev V.A., Mihelic E.Z., Benko E., Budylowski P., Grocott S., Lee T., Korosec C.S., Colwill K., Stephenson H., Law R. (2023). Immunogenicity of COVID-19 vaccines and their effect on HIV reservoir in older people with HIV. iScience.

[24] Murata M., Matsumoto Y., Shimono N. (2025). Comparison of SARS-CoV-2 antibody responses following the second dose of BNT162b2 and mRNA-1273 vaccines in people living with HIV-1. Vaccine.

[25] Lahdentausta L., Kivimaki A., Oksanen L., Tallgren M., Oksanen S., Sanmark E., Salminen A., Geneid A., Sairanen M., Paju S. (2022). Blood and saliva SARS-CoV-2 antibody levels in self-collected dried spot samples. Med. Microbiol. Immunol..

[26] Klingler J., Rao P.G., Bandres J.C., Pena I., Roldan K.B., Singh G., Monahan B., Gleason C., Chen Y., Slamanig S. (2025). Reduced Fc-mediated antibody responses after COVID-19 mRNA vaccination in a cohort of people living with HIV-1. Sci. Rep..

[27] Cerutti A. (2008). The regulation of IgA class switching. Nat. Rev. Immunol..

[28] Twigg H.L., Spain B.A., Soliman D.M., Bowen L.K., Heidler K.M., Wilkes D.S. (1996). Impaired IgG production in the lungs of HIV infected individuals. Cell. Immunol..

[29] Cognasse F., Beniguel L., El Habib R., Sabido O., Chavarin P., Genin C., Garraud O. (2003). HIV-gp160 modulates differentially the production in vitro of IgG, IgA and cytokines by blood and tonsil B lymphocytes from HIV-negative individuals. Clin. Exp. Immunol..

[30] Spickett G.P., Dalgleish A.G. (1988). Cellular immunology of HIV-infection. Clin. Exp. Immunol..

[31] Navas A., dos Santos J.C., van Cranenbroek B., Vadaq N., Groenendijk A.L., Vos W.A.J.W., Blaauw M.J.T., van Eekeren L., Rokx C., Stalenhoef J.E. (2025). Circulating immune landscape and immune signatures in spontaneous HIV controllers. Front. Immunol..

[32] Paul M.J., Hudda M.T., Pallett S., Groppelli E., Boariu E., Finardi N.F., Wake R., Sofat N., Biddle K., Koushesh S. (2025). Mucosal immune responses to SARS-CoV-2 infection and COVID-19 vaccination. Vaccine.

[33] Marcotte H., Cao Y., Zuo F., Simonelli L., Sammartino J.C., Pedotti M., Sun R., Cassaniti I., Hagbom M., Piralla A. (2024). Conversion of monoclonal IgG to dimeric and secretory IgA restores neutralizing ability and prevents infection of Omicron lineages. Proc. Natl. Acad. Sci. USA.

[34] Pisanic N., Antar A.A.R., Hetrich M.K., Demko Z.O., Zhang X., Spicer K., Kruczynski K.L., Detrick B., Clarke W., Knoll M.D. (2025). Early, Robust Mucosal Secretory Immunoglobulin A but not Immunoglobulin G Response to Severe Acute Respiratory Syndrome Coronavirus 2 Spike in Oral Fluid Is Associated with Faster Viral Clearance and Coronavirus Disease 2019 Symptom Resolution. J. Infect. Dis..

[35] Lofstrom E., Eringfalt A., Kotz A., Weckbom F., Tham J., Lingman M., Nygren J.M., Unden J. (2021). Dynamics of IgG avidity and antibody levels after COVID-19. J. Clin. Virol..

[36] Manuylov V., Burgasova O., Borisova O., Smetanina S., Vasina D., Grigoriev I., Kudryashova A., Semashko M., Cherepovich B., Kharchenko O. (2022). Avidity of IgG to SARS-CoV-2 RBD as a prognostic factor for the severity of COVID-19 reinfection. Viruses.

[37] Lara-Aguilar V., Llamas-Adan M., Brochado-Kith O., Crespo-Bermejo C., Grande-Garcia S., Arca-Lafuente S., de Los Santos I., Prado C., Alia M., Sainz-Pinos C. (2024). Low-level HIV-1 viremia affects T cell activation and senescence in long-term treated adults in the INSTI era. J. Biomed. Sci..

[38] Shive C.L., Freeman M.L., Younes S., Kowal C.M., Canaday D.H., Rodriguez B., Lederman M.M., Anthony D.D. (2021). Markers of T cell exhaustion and senescence and their relationship to plasma TGF-β levels in treated HIV+ immune non-responders. Front. Immunol..

[39] Naismith E., Pangrazzi L., Grasse M., Keller M., Miggitsch C., Weinberger B., Trieb K., Grubeck-Loebenstein B. (2019). Peripheral antibody concentrations are associated with highly differentiated T cells and inflammatory processes in the human bone marrow. Immun. Ageing.

[40] Moosa A., Gengiah T.N., Lewis L., Naidoo K. (2019). Long-term adherence to antiretroviral therapy in a South African adult patient cohort: A retrospective study. BMC Infect. Dis..

[41] Bijker R., Kiertiburanakul S., Kumarasamy N., Pujari S., Sun L.P., Ng O.T., Lee M.P., Choi J.Y., Nguyen K.V., Chan Y.J. (2020). Survival after long-term ART exposure: Findings from an Asian patient population retained in care beyond five years on ART. Antivir. Ther..

