# Supplementary material for: Mucosal Immune Responses in People Living with HIV May Confer Protection from SARS-CoV-2 Infections After COVID-19 Vaccination
Source: Vaccines (Basel). 2026 May 30;14(6):493. doi: 10.3390/vaccines14060493 (PMC13308110; doi:10.3390/vaccines14060493)
Supplement: Supplementary file 1 [file vaccines-14-00493-s001.zip › vaccines-4311120-supplementary.pdf]

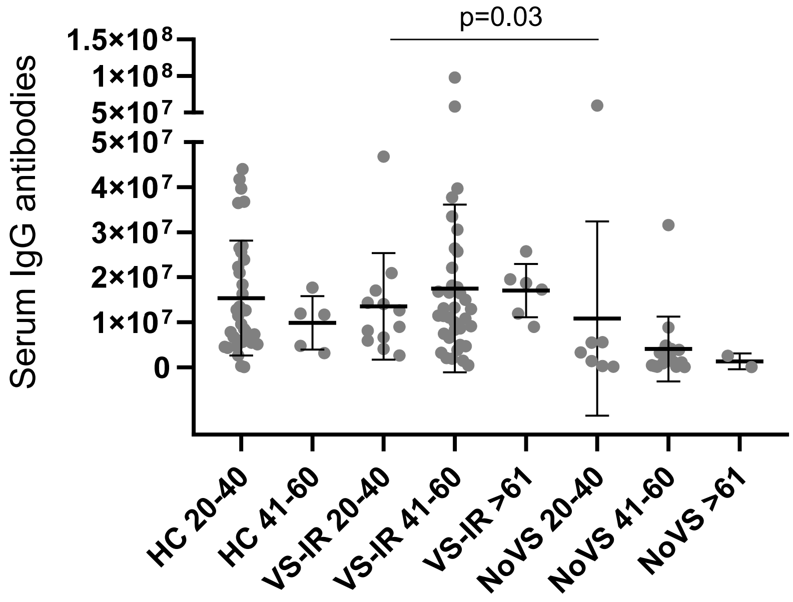

Figure S1. Age-wise stratification of serum anti-SARS-CoV-2 spike IgG antibodies. The circles denote the samples and the error bars denote mean ± SD. HC – HIV-negative healthy control; VS-IR – PLWH with viral suppression and Immunological reconstitution; NoVS – PLWH without viral suppression.

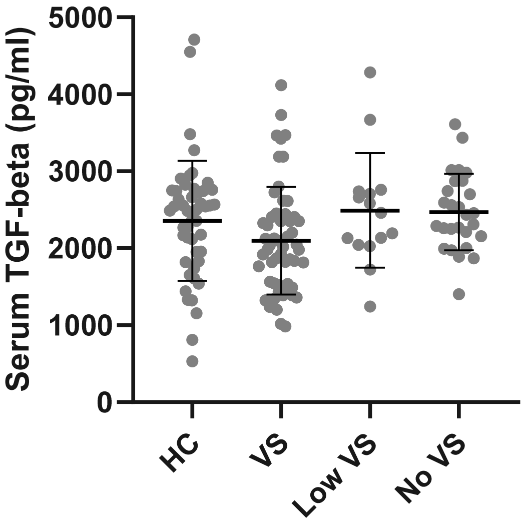

Figure S2. Serum TGF-β levels (pg/ml) in HIV-negative healthy controls (HC) and people living with HIV (PLWH) cohorts. The circles denote the samples and the error bars denote mean±SD. VS – PLWH with viral suppression; Low VS – PLWH with low HIV RNA copies; NoVS – PLWH without viral suppression.

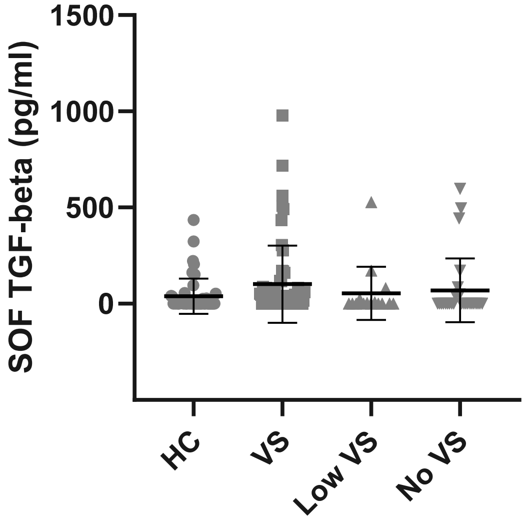

Figure S3. Stimulated oral fluid (SOF) TGF-β levels (pg/ml) in HIV-negative healthy controls (HC) and people living with HIV (PLWH) cohorts. The circles denote the samples and the error bars denote mean±SD. VS – PLWH with viral suppression; Low VS – PLWH with low HIV RNA copies; NoVS – PLWH without viral suppression.
